# Effects of UV-C and Edible Nano-Coating as a Combined Strategy to Preserve Fresh-Cut Cucumber

**DOI:** 10.3390/polym13213705

**Published:** 2021-10-27

**Authors:** María L. Zambrano-Zaragoza, David Quintanar-Guerrero, Ricardo M. González-Reza, María A. Cornejo-Villegas, Gerardo Leyva-Gómez, Zaida Urbán-Morlán

**Affiliations:** 1 Laboratorio de Procesos de Transformación y Tecnologías Emergentes de Alimentos, UNAM, FES-Cuautitlán, Cuautitlan Izcalli Z.P. 54714, Mexico; gonzalez.reza@comunidad.unam.mx (R.M.G.-R.); angiecornejo@unam.mx (M.A.C.-V.); 2 Laboratorio de Posgrado en Tecnología Farmacéutica, Universidad Nacional Autónoma de México, FES-Cuautitlán, Cuautitlan Izcalli Z.P. 54740, Mexico; quintana@unam.mx; 3 Departamento de Farmacia, Facultad de Química, Universidad Nacional Autónoma de México, Ciudad de Mexico Z.P. 04510, Mexico; leyva@quimica.unam.mx; 4 Centro de Información de Medicamentos, Facultad de Química, Universidad Autónoma de Yucatán, Mérida Z.P. 97069, Mexico; zaida.urban@correo.uady.mx

**Keywords:** alginate-pectin, nanoparticles, lemon essential oil, antioxidant capacity, emerging technologies

## Abstract

The objective of this study was to evaluate the effectiveness of a combination of UV-C disinfection treatment and a nano-coating lemon essential oil nanocapsules. The nanocapsules were prepared by ionic gelation with an alginate-pectin wall and the lemon essential oil had a particle size of 219 ± 22 nm and a zeta potential of −7.91 ± 0.18 mV. The lemon essential oil had an encapsulation efficiency of 68.19 ± 1.18%. The fresh-cut cucumber was stored for 15 days at 4 °C. Six formulations of nanocapsules were evaluated, and hydroxypropyl methylcellulose was used as matrix polysaccharide in four coatings. Three formulations were treated with UV-C at 4.5 kJ/m^2^. The results showed that the combination of UV-C and nano-coatings (lemon essential oil = 200 mg/L) increased the shelf life by up to 15 days. Using UV-C and nano-coatings, the ∆E value was 7.12 at the end of the storage period, while the Control samples had an ∆E of 28.1. With nano-coating treatment, the amount of polyphenols decreased by 23% within 9 days. In contrast, with combined UV-C and nano-coating treatment, the amount of polyphenols was reduced by 38.84% within 15 days. The antioxidant capacity remained stable at 459 μmol TE/100 g for the fresh product when the combined treatment was used. A good correlation was also observed between the increasing of the fruit’s shelf life and decreasing of its enzymatic activity. The inclusion of UV-C treatment contributed to the reduction in the initial total bacteria at 3.30 log CFU/g and its combination with nano-coatings helped in the control of microbial growth during storage.

## 1. Introduction

In today’s world, many consumers prefer fresh foods because of their greater nutritional value. In order for beneficial health effects to be achieved, however, these foods must maintain good physicochemical and sensory quality, as well as being safe for consumption and free of contaminants and pathogenic microorganisms [1]. Cucumber (*Cucumis sativus* L.) is a very popular fruit worldwide due to its organoleptic characteristics and its crisp, fresh taste, while its high nutritional value, which is due to its vitamin, nicotinic acid, and mineral contents, favors consumer health [2]. Cucumber’s high moisture content, however, presents a challenge for storage, even under refrigeration conditions, because it is susceptible to increased respiration, yellowing, moisture loss, and increased microbial activity, which generates decay, and thus, reduces its shelf life. Thus, it is necessary to protect this fruit with technologies that reduce post-harvest losses [3]. In this regard, significant progress has been made in developing edible nano-coatings [4,5]. Modifying permeability to O_2_ and CO_2_ and adding active components that are capable of improving such properties as antioxidant and antimicrobial capacity can considerably reduce enzymatic and respiratory activity and, therefore, increase the shelf life of fresh-cut, ready-to-eat products [6]. Hashemi et al. (2020) [7] affirmed that an active coating helps keep food safe and preserve sensory properties, as in the case of strawberries stored at 4 °C, where the formation of a thin membrane produced by a film-forming dispersion helped maintain firmness [8].

These types of coatings can contain polysaccharides, proteins, lipids, or combinations of these substances. One good option is hydroxypropyl methylcellulose (HPMC) since it reduces the respiration rate and increases the shelf life of fruits [6]. An active substance such as lemon essential oil (LEO) can be encapsulated into nanostructured systems and then be incorporated into coatings to increase the shelf life of cucumbers [9]. One acceptable, polyfunctional encapsulant material is sodium alginate, a carbohydrate derived from algae that consists of α-L-mannuronic acid and β-D glucuronic acid. This water-soluble and non-toxic food additive is used to maintain the quality of fresh-cut products during storage by inhibiting fungal growth [10]. Applying edible coatings with essential oils on fresh-cut products is an attractive strategy considering their natural origin and antioxidant and antimicrobial properties, all of which add value [11]. LEO has been used in fresh-cut products since research showed that some bacteria are susceptible to its components (e.g., D-limonene) [9]. UV-C radiation treatments, meanwhile, have been used as an alternative to heat processes for reducing microorganisms, and thus, conserving the quality of fresh-cut fruits and vegetables [1].

Our group recently reported that alginate-pectin nanoparticles (NP) prepared by ionic gelation with various essential oils, including LEO, were stable and presented significant antioxidant properties that are beneficial for food preservation [12]. Olawuyi and Lee (2019) [3] evaluated the quality of fresh-cut cucumbers after coating with chitosan and packaging in various polymer films. They found that a 2% chitosan coating was efficient in maintaining product quality. However, observations also show that treatment efficiency is a function of the specific film selected for packaging. Fan et al. (2020) [2] studied the effect of ultrasound and an algae-based coating on fresh-cut cucumbers. Although they found that shelf life could be prolonged to 15 days, this mode of treatment proved expensive and unsuitable for consumers with algae allergies. 

The hormesis produced by abiotic stress is an alternative to reduce the microbial growth and increase the shelf life of fresh-cut fruits, and the hormeses caused by the use of low doses of UV-C irradiation in these products has been reported to the produce beneficial effects in terms of the modification and synthesis of polyphenols; this treatment has been extensively investigated [13,14]. Recent research includes advances in developing nano-coatings based on emerging technologies, but little information is available on the effect of combined strategies in terms of preserving the quality of fresh fruit during storage; for example, the effects surface disinfection with UV-C radiation accompanied by a nano-coating have not been adequately investigated. The main aim of this study was to evaluate the combined effect of an HPMC nano-coating containing LEO-alginate-pectin (NP) prepared by ionic gelation with UV-C radiation as a new approach proposed to increase shelf life, preserve fruit quality, and decrease enzymatic activity in fresh-cut cucumbers during storage at 4 °C.

## 2. Materials and Methods

### 2.1. Materials

Lemon essential oil, citrus pectin (galacturonic acid 65%), and sodium alginate (mannuronic/guluronic ratio of 1.6 Mw ~216 g/mol from brown algae) were purchased from Drogueria Cosmopolita S.A. de C.V. (Mexico City, MX, USA). Tween^®^80, Span^®^80, and radical DPPH (2,2-diphenyl-1-picrylhydracil) were purchased from Sigma Aldrich^®^ (St. Louis, MI, USA). Polyvinyl alcohol (PVA) (Mowiol^®^ Mw ~26 kDa, Glo-Marza, México City MX, USA) was used as the stabilizing agent. Distilled water was of Milli-Q^®^ grade (Millipore Corporation, Bedford, MA, USA). All other reagents were of analytical grade.

### 2.2. Cucumbers

The cucumbers were obtained from a supply center in the State of Mexico (Central supply center, Cuautitlan, MX, USA). They were selected in the stage of physiological maturity with protrusions in the pericarp, were free of mechanical, microbial, or physiological damage, and were of uniform size and green color. They were washed, disinfected in a solution of 100 ppm of sodium hypochlorite, peeled, and cut to apply the corresponding treatment.

### 2.3. Preparation of Lemon Essential Oil Nanoparticles

Lemon essential oil alginate-pectin nanoparticles (LEO-A-P NP) were prepared by the ionic gelation method. Briefly, a nanoemulsion was prepared with 2 g/L of LEO using Span^®^80 and PVAL 1:1 as stabilizers. The process was carried out in two homogenization cycles at 1413.7 s^−1^ for 5 min using a rotor-stator system (Ultra-Turrax^®^ T25 Digital, IKA, Staufen, Germany). The NP were prepared using 40 mL of polysaccharides with 2.5 g/L of sodium alginate, 5 g/L of citric pectin, and 20 mL of the nanoemulsion. The obtained system was placed in a syringe pump (Syringe Pump, New Era Pump Systems, Inc., Farmingdale, NY, USA) and dripped at a rate of 0.2 mL/min into 10 mL of a solution of ZnCl_2_ 0.0156 mol and 10 mL of PVAL at 5 g/L to cross-link the particles. The dispersion was then homogenized in 3 cycles at 1047.2 s^−1^/3 min (Ultra-Turrax^®^ T25 Digital, IKA, Staufen, Germany).

### 2.4. Characterization of the LEO-A-P-NP

The particle size (PS) and the polydispersity index (PDI) were determined at a 90° angle by laser-scattering using a Z-sizer 4 (Zetasizer Nano Series, Malvern Ltd., Enigma Business Park, Grovewood Road, UK). The samples were prepared by diluting 1 mL of the nanoparticle dispersion in 9 mL of Milli^®^ Q distilled water. The zeta potential (ζ) was determined by doppler laser microelectrophoresis (He/Ne laser beam 633 nm in length and 4 mW) of the dispersed particles using the same equipment. All determinations were performed in triplicate at 25 °C.

### 2.5. Encapsulation Efficiency (EE)

The encapsulation efficiency of the nanoparticles was determined by sedimenting them with a Hermle Z323K centrifuge (Labortechbnik GMBH, Wehingen, Germany) at 21,420× *g* for 30 min at 4 °C. The supernatant was separated to determine the amount of non-encapsulated lemon oil and the sample was analyzed for absorbance at λ = 265 nm on a UV-Vis Genesys 10 s UV/Vis spectrophotometer (Thermo Fisher Scientific Inc., Waltham, MA, USA). All determinations were performing in quintuplicate, and the EE was obtained by the following equation:

(1) $$\mathrm{EE}\;\left(\%\right)=100-\frac{\mathrm{LEO}\;\mathrm{in}\;\mathrm{supernatant}}{\mathrm{initial}\;\mathrm{LEO}\;\mathrm{concentration}}\times 100$$

### 2.6. Preparation of the Coating Dispersion

The film-forming dispersion was prepared using HPMC at 10 g/L, which was dispersed at 80 °C for 20 min with constant stirring. Propylene glycol at 10 g/L was used as a plasticizer. The nano-coating was prepared using the LEO-A-P NP at an LEO-equivalent concentration of 200 mg/L in the final HPMC dispersion.

### 2.7. UV-C Irradiation Conditions and Application of the Coating

The dose of irradiation applied to the fresh-cut cucumbers was selected after conducting preliminary studies (not shown) to establish the optimal level in terms of the changes observed in color and subjective appearance. UV-C treatment was carried out in a chamber with aluminum-coated walls using a 15-W UVP lamp model XX-15S at 254 nm (Steril-air lamp, Burbank, CA, USA). The samples were placed at 30 cm from the lamp. The intensity of the UV-C light emitted was measured by a Lutron Model RS-232 photo-radiometer (Lutron Electronic Enterprise Co., LTD., Taipei, Taiwan). The radiation intensity was the average of 10 measurements. The dose applied was 4.5 kJ/m^2,^ as a function of exposure time and distance [15].

### 2.8. Application of the Nano-Coating and Packaging of the Cucumber

To evaluate the combined effect of the UV-C irradiation treatment with the nano-coating, 6 different batches were prepared: (1) Control; (2) HPMC; (3) HPMC-LEO-A-P-NP; (4) UV-C-Control; (5) UV-C-HPMC; and (6) UV-C- HPMC-LEO-A-P NP. The film-forming dispersions were applied to each batch by immersion for 3 min, then 140 g of cucumber from each treatment were packed in polypropylene trays and stored at 4 °C. All samples were prepared in triplicate.

### 2.9. Headspace Gas Analysis

The concentration of gases inside the packaging was measured with a Quantek^®^ Instruments model 902D gas analyzer Dualtrack (Quantek Instruments, Inc., Grafton, MA, USA). Prior to packaging, a septum was placed in each container to prevent gas leaks due to perforation during the measuring process. The concentrations of O_2_ and CO_2_ were measured every third day from day 0 to day 15. All measurements were performed in triplicate to determine the composition of gases in the headspace.

### 2.10. Weight Loss, Total Soluble Solids (TSS), and pH

Weight loss was determined by measuring the weight difference. Initial weights were recorded before storage, then the samples were weighed on a digital scale every third day up to day 15. To perform this determination of TSS and pH, the fresh-cut cucumbers were first homogenized. TSS were determined in a Hanna model HI 96801 digital refractometer (Hanna Instruments, Woonsocket, RI, USA). pH was measured directly by placing homogenized cucumber inside the electrode of a model PH213 Hanna potentiometer (Hanna Instruments, Woonsocket, RI, USA). All determinations were performed in triplicate.

### 2.11. Firmness and Color

Firmness was determined using a Brookfield model CT3 Texturometer (AMETEK Brookfield Inc. Chandler, AZ, USA) with a load cell of 25 kg. A 6 mm diameter plane fixed puncture (TA41) was applied to 3 mm of the surface of the cucumber. The activation load was 0.1 N, and the probe rate was 1.5 mm/s. The top peak in the graphical distance (mm) vs Force (N) represents the firmness of the sample. All determinations were performed in quintuplicate. 

Color measurement was carried out in a CM-600d spectrophotometer (Konica Minolta Sensing Americas, Inc. Ramsey, NJ, USA), with the D65 illuminant at 10° from the observer after calibration with a standard white plate. The L*, a*, and b* values obtained were used to calculate the yellowing index (YI) and total color difference (∆E) according to the Equations (2) and (3).

(2) $$\mathrm{Yellowing}\;\mathrm{index}\;\left(\mathrm{YI}\right)=142.48\frac{b}{L}$$

(3) $$\Delta E=\sqrt{{\left(L-L_{0}\right)}^{2}+{\left(a-a_{0}\right)}^{2}+{\left(b-b_{0}\right)}^{2}}$$

where ∆E values of 0.5–1.5 represent slight differences; ∆E values of 1.5–3.0 represent noticeable differences; ∆E values of 3.0–6.0 represent appreciable differences; ∆E values of 6.0–12.0 represent large differences; and ∆E values > 12 represent obvious differences [16].

### 2.12. Chlorophyll Content

Chlorophyll content was determined following the methodology proposed by Zhang et al. (2008) [17]. Briefly, 5 g of cucumber were homogenized in 20 mL of acetone–water solution (80:20) using an IKA^®^ ultraturrax homogenizer (IKA^®^ WERKE, Staufen, Germany). This mixture was centrifuged at 11,088× *g* at 4 °C/20 min in a Hermle Z323K centrifuge (Labortechnik GMBH, Wehingen, Germany). The supernatant was filtered using a PTFE syringe filter with 0.45 µm pores. Absorbance was measured at λ = 647 nm in a Genesys 10 s UV/Vis spectrophotometer (Thermo Fisher Scientific Inc., Waltham, MA, USA). Chlorophyll content was calculated using the following equation:

(4) $$\mathrm{Chlorophyll}\;\left(\frac{\mathrm{mg}}{g}\right)=17.95A_{647}+7.9A_{665}$$

### 2.13. Ascorbic Acid

Ascorbic acid (AA) was determined by the titration method with 2,6-dichloroindophenol-indophenol. The dye factor of the indophenol solution was obtained before analyzing the sample [18]. Determination of AA was carried out by homogenizing 10 g of cucumber with a 3% phosphoric acid solution gauged to 100 mL using a biobased BK-HG160 homogenizer (Biobase, Focus Technology Co., Ltd., Jiangsu, China). Subsequently, this mixture was centrifuged at 11,088× *g* for 20 min at 4 °C. The extract obtained was filtered, then 5 mL were taken and titrated with 2,6-dichloroindophenol until the mixture turned pink. All determinations were performed in triplicate. The result is expressed as g of AA/100 g of fresh cucumber.

### 2.14. Total Phenol Content (TPC)

TPC was determined by means of the Folin–Ciocalteu colorimetric method adapted by [16]. Briefly, 10 g of cucumber homogenized with 20 mL of methanol were used to obtain the extract, which was stirred for 1 h under darkness. The sample was centrifuged at 11,088× *g*/20 min at 4 °C. The supernatant represented the phenol extract. This was separated and filtered with a polytetrafluoroethylene syringe filter (pore size 0.45 µm). Next, 250 µL of Folin reagent were incorporated into 20 µL of extract. After 8 min, 750 µL of Na_2_CO_3_ and 950 µL of distilled water were added. This mixture was incubated for 30 min and absorbance was measured at λ = 765 nm in a UV-Vis Genesys 10 s spectrophotometer. All determinations were performed in triplicate. The results are expressed as mg of gallic acid equivalent (mg EAG/100 g of cucumber).

### 2.15. Radical Scavenging Activity (DPPH)

DPPH activity was determined according to the methodology followed by [19]. The same extract used to determine polyphenols was utilized to measure DPPH. One 100-µL aliquot of extract and 2.9 mL of DPPH (240 mg/L in methanol) solution were maintained under agitation for 30 min in the dark. Absorbance was measured at λ = 515 nm. The results are expressed as the µmol Trolox Equivalent/100 g of fresh cucumber.

### 2.16. Enzymatic Activity

Three enzymatic evaluations—peroxidase, pectin methylesterase, and polyphenol esterase activity—were performed to indicate any changes in, or influence on, the quality parameters of the fruit.

#### 2.16.1. Peroxidase Activity (POD)

POD activity was determined by measuring the change in absorbance during 1 min at λ = 470 nm, according to the method proposed by [20]. Briefly, cucumber was mixed with a 2-mol solution of NaCl and polyvinylpyrrolidone (10 g/L), and homogenized in a BK-HG160 homogenizer (Biobase, Shandong, China) for 10 min. The mixture was centrifuged at 11,088× *g*/30 min. The supernatant was filtered through a polytetrafluoroethylene syringe filter with a pore size of 0.45 µm. Enzymatic activity was determined by mixing 1 mL of guaiacol (1%), 1 mL of H_2_O_2_ (1%), and 50 µL of extract. Changes in absorbance were recorded at λ = 470 nm for 3 min. Enzyme activity was calculated based on the linear portion of the curve with respect to time. One unit of guaiacol (U) was defined as the amount of enzyme that oxidizes 1 µmol of guaiacol per minute at 25 °C.

#### 2.16.2. Pectin Methylesterase (PME)

The enzymatic extract was prepared with 30 g of fresh-cut cucumber in 30 mL of 0.2-mol NaCl solution and 10 g/L of polyvinylpyrrolidone. The mixture was stirred for 10 min in an ice bath and the homogenate was centrifuged at 11,088× *g* at 4 °C/40 min. The supernatant was filtered through a PTFE syringe filter. The solution obtained was the enzymatic extract used to determine PME. The solution was adjusted to pH = 7.5 with 2 mol or 0.2 mol of NaOH. PME activity was determined following the methodology proposed by [21], which meant measuring the decrease in absorbance at λ = 640 nm for 3 min.

#### 2.16.3. Polyphenol Oxidase (PPO)

PPO activity was determined according to the methodology proposed by [16]. Briefly, 0.2 mL of enzymatic extract was mixed with 2.8 mL of catechol (50 mmol in 0.2-mol phosphate buffer at pH 6.5). The change in relative absorbance was compared to a white-without-enzyme extract and measured at λ = 420 nm in a UV-Vis Genesys spectrophotometer for 10 s. One unit of PPO activity was identified as the change of 0.001 abs/min obtained from the initial slope. All determinations were made in triplicate.

### 2.17. Microbiological Analyses

MC-Media pad plates (Merck KGaA, Darmstadt, Germany) were used to determine the growth of bacteria, fungi, and yeast. In this step, 10 g of cucumber were homogenized aseptically in 100 mL of physiological saline solution for 2 min. Dilution series were prepared beginning at 10^−2^ for fungi and yeast, and 10^−3^ for aerobic bacteria. The pad plates for aerobic bacteria were incubated at 35 °C/24 h, while the ones for fungi and yeast were incubated at 25 °C/72 h. The results are expressed as CFU/g. All determinations were performed in triplicate.

### 2.18. Statistical Analyses

Statistical analyses were performed using Minitab^®^ 18 software (Minitab^®^ Statistical Software 18 Inc., Centre, PA, USA). The variance (ANOVA) analysis was determined considering a *p* value ≤ 0.05. A Tukey test was used to determine the differentiation of the means.

## 3. Results

### 3.1. Characterization of the Alginate-Pectin Nanoparticles

The LEO-A-P-NP had an average particle size of 219 ± 0.215 nm with a narrow distribution (PDI 0.29 ± 0.02). These results coincide with those obtained under the same conditions by [12] and are consistent for alginate nanoparticles cross-linked with ZnCl_2_ [22]. The ζ was negative at −7.91 ± 0.18 mV, a finding that can be related to the thick steric layer of PVAL on the NP surface and the electrokinetic effect of carboxyl groups of the alginate phenol groups in essential oils [23]. The encapsulation efficiency was 68.19 ± 1.18%, which can be considered high since this parameter was previously reported to be under 55% when the concentration of essential oil was at a ratio of 10:0.4 (*w*/*w*) [10]. In our case, the polymeric wall prepared with alginate-pectin led to entrapment of the essential oil (2 g/L).

### 3.2. CO_2_ Concentration in Fresh-Cut Cucumber

**Figure 1:**
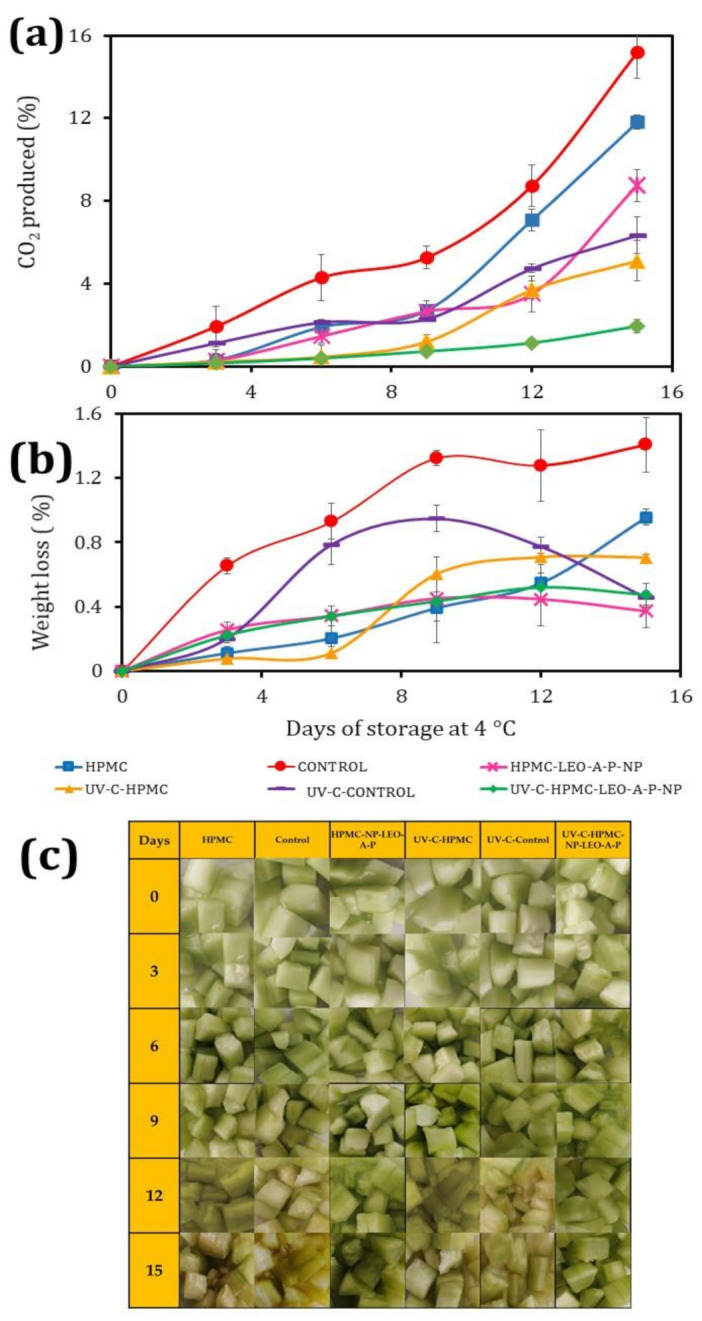
Changes of CO_2_, weight loss and visual evolution during storage of fresh-cut cucumber at 4 °C: (**a**) CO_2_ evolution on headspace, (**b**) weight loss, and (**c**) visual changes.

Figure 1a shows the CO_2_ changes in the fresh-cut cucumber during storage of the different treatments. The control samples had increased CO_2_ concentrations from day 3, which was associated with physiological changes that increased the respiration rate, microbial growth, tissue breakdown, and the release of cell content, all of which greatly reduce shelf life. By day 9, the deterioration was considerable, indicating a more significant increase in CO_2_ production. It is important to emphasize that the HPMC-LEO-A-P-NP showed no statistically significant difference at day 9, though this increased at day 12, with this increase being associated with microbial growth. The use of polysaccharide-based coatings reduced the respiration rates. This nano-coating presents an excellent barrier to gases and provides control of physiological changes during storage. Other authors have reported that they effectively control the respiration and deterioration of fresh-cut fruits [24]. The HPMC-LEO-A-P-NP coating controlled the respiration rate up to day 12, and the results indicate that the LEO contributed to decreasing the respiration rate and particle size of the NP, while also providing a homogeneous distribution over the entire surface area. In addition, it controlled the release of LEO, thus helping to generate a modified atmosphere [9]. In the UV-C-Control treatment, CO_2_ remained stable up to day 9, while the UV-C-HPMC and UV-C-HPMC-LEO-A-P-NP batches maintained constant CO_2_ production. This behavior was associated with a delay in microbial growth. Other authors have also reported that HPMC-based coatings help to decrease the rate of gas exchange [25,26]. When the combined UV-C-HPMC-LEO-A-P-NP treatment was applied, improved product stability was obtained up to day 15, demonstrating the advantages of using both procedures, that is, disinfection with UV-C plus the nano-coating with 200 mg of LEO.

### 3.3. Weight Loss

Figure 1b shows the weight loss of the treated fresh-cut cucumbers compared to the untreated Control sample. The Control sample had a weight loss of 1.4%, which was better than the result obtained for fresh-cut cucumber without coating placed in a modified atmosphere of argon or air, where losses were as high 5% in Controls [26]. The HPMC-NP-LEO-A-P with, and without, UV-C treatment had weight losses below 0.5% after 15 days of storage at 4 °C. These results were better than those obtained for fresh-cut cucumbers coated with chitosan at 1 and 2% and packed in polypropylene clamshells. In that case, the author recorded losses of 1–2% [3]. The use of HPMC-LEO-A-P-NP thus decreased weight loss compared to chitosan nanoparticles made with cinnamon essential oil, where the loss was 5.49% after 16 days of storage [27]. The weight loss was best controlled on all samples with nano-coating. The particle size was crucial because greater surface areas enhance the capacity to trap water between fruit and coating, thus reducing the weight losses associated with the drained liquid [28]. Figure 1c shows the visual changes on fresh-cut cucumber during storage at 4 °C, evidencing the positive effect of using LEO nanocapsules and highlighting that the initial treatment with UV-C (UV-C HPMC-LEO-A-P-NP) contributes to the slowing down of the deterioration reaction’s speed, which is associated with enzymatic and microbiological changes. According to the image in Figure 1c the Control samples kept their characteristics for only 6 days, as compared with the samples with HPMC-LEO-A-P-NP that retained their visual aspects almost 12 days and the samples with UV-C and HPMC-LEO-A-P-NP that had a shelf life of 15 days.

### 3.4. Total Soluble Solids and pH

Table 1 shows the changes in soluble solids during storage, where minimal variations are visible among treatments, with these variations initially being smaller in the HPMC-LEO-A-P-NP samples due to the incorporation and absorption of the nanosystems into the tissue. The differences on the ensuing days were not statistically significant (*p* ≤ 0.05). The UV-C-HPMC treatment showed more significant variations during storage, which were associated with polysaccharide modifications under UV-C treatment. These slight changes may also be related to the water concentration, as this can cause a more significant dilution of TSS over time due to evaporation. The increase in TSS may also be attributable to the degradation of sugars through the action of microorganisms [29]. Table 1 shows the change in pH as a function of treatment. Here, a significant statistical difference (*p* ≤ 0.05) was found depending on time and the specific treatment used, though no statistically significant difference in pH could be attributed to UV-C treatment. At the same time, the combined UVC-HPMC coating helped to maintain the pH values during storage. It is important to note that the NP made with LEO contributed even more greatly to controlling the pH, as this generally tended to increase with greater storage time. The increase in these samples coincides with that reported by other authors for fresh-cut cucumber coated with chitosan at 1 and 2% in the sense that the coating helped to control the pH [3], These reaffirmed the positive effect of the coatings on parameters such as TSS and pH, which was associated with the water absorption capacity of the HPMC used as a matrix polymer, thereby retaining soluble solids and pH.

### 3.5. Chlorophyll

**Figure 2:**
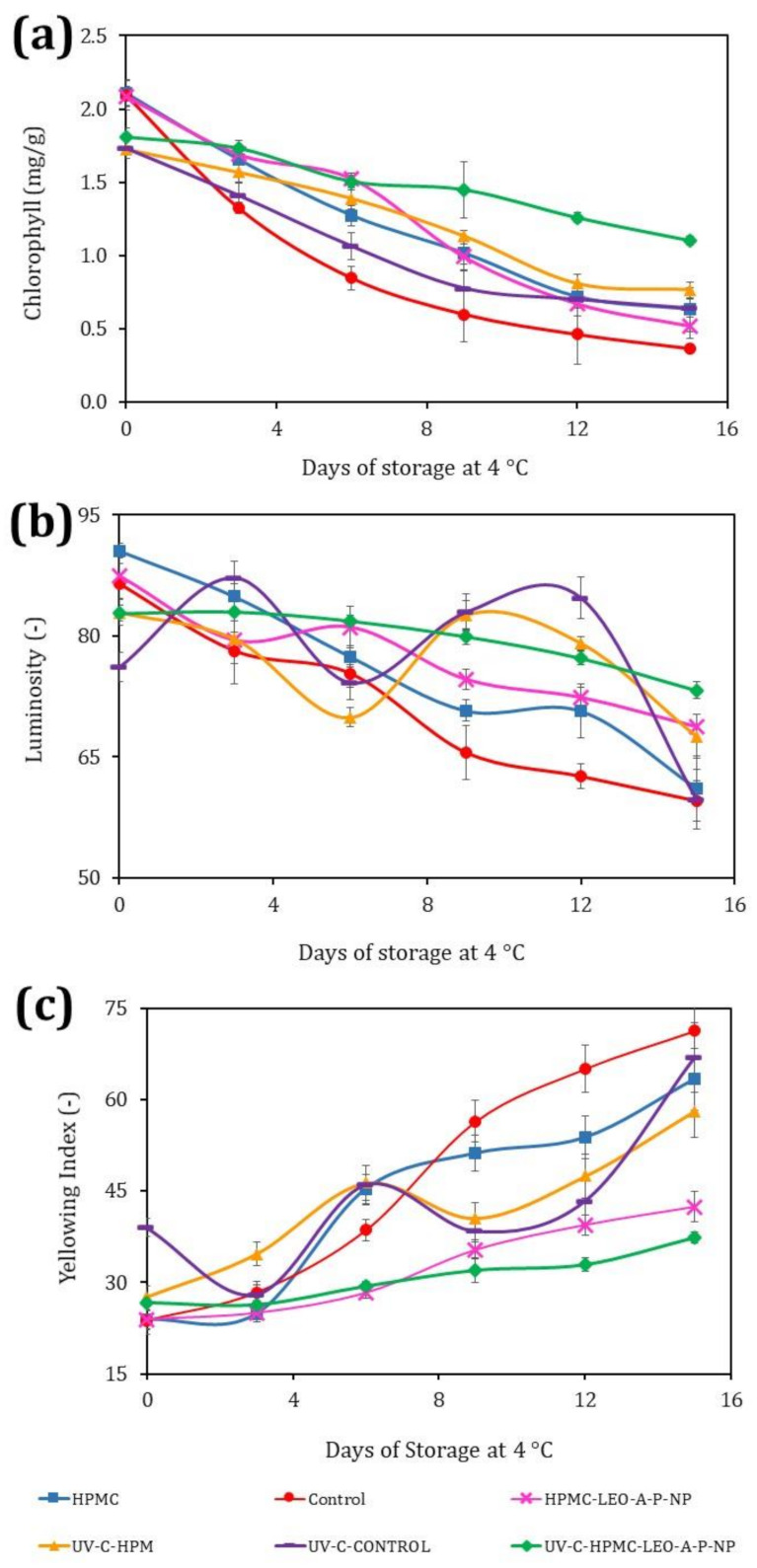
(**a**) Chlorophyll changes in fresh-cut cucumber at 4 °C (mg/g); (**b**) luminosity changes for the different treatments during storage at 4 °C; and (**c**) yellowing index of fresh-cut cucumber.

Fresh-cut cucumber is attractive to consumers because of its turgor and light-green color. Chlorophyll plays an essential role in the visual quality of this product since degradation leads to yellowing, which reduces commercial quality [30]. Figure 2a shows the changes in chlorophyll content, which decreased as a function of time in all cases. A study of broccoli florets at a dose of 4.5 kJ/m^2^ significantly reduced initial chlorophyll content (16.3%), which was attributable to the irreversible breakdown of chlorophyll [31]. The UVC-HPMC-LEO-A-P-NP helped to stabilize chlorophyll in the fresh-cut cucumber with a 39% decrease at day 15 compared to the same treatment without UV-C, where the reduction was as high as 70% of the initial chlorophyll content. It is important to point out that the HPMC-LEO-A-P-NP formulation acts effectively in terms of reducing the loss of chlorophylls due to its antioxidant capacity, whereas UV-C increases the production of reactive oxygen species (ROS). Then, the combination of treatment contributes effectively to maintain the stability of chlorophylls. [32]. This coating offers an economical, easy-to-apply technology that gives results as good as those reported for fresh-cut cucumber exposed to a pasteurized argon atmosphere, where the chlorophyll reduction was 26% after 12 days [1]. The HPMC-LEO-A-P-NP coating only had a decrease in chlorophyll of 26% at day 9, and thus, this coating helped to maintain the stability of the chlorophyll. Moreover, it did not show a statistically significant difference compared to the fresh-cut cucumber with the UVC-HPMC-LEO-A-P-NP treatment. The reduction in chlorophyll in the samples treated with UV-C is associated with changes in the synthesis of secondary metabolites that occur as defense mechanisms generated by cells under this condition [33].

### 3.6. Color of the Fresh-Cut Cucumber

Figure 2b shows the changes in brightness with evident and statistically significant differences (*p* ≤ 0.05) among treatments. The Control had a slight increase in brightness at 6 days associated with the breakdown of cellular structures, though this later decreased. The UV-C-Control showed a decrease up to day 9, but luminosity later increased, before descending again at the end of storage. This behavior was attributed to cell breakdown and tissue decomposition due to the microbial growth and enzymatic activity [33].

Similar results were reported for minimally processed sliced apples treated with UV-C (1.2–24 kJ/m^2^) as, after 15 days of storage, an increase in luminosity was observed that indicated the rupture and cytoplasmic expulsion of components [34]. The HPMC-coated cucumber had a decrease in luminosity of 31.7% at the end of storage, suggesting a beneficial effect of this Control. After 6 days, there was also a drop of 15% in visual quality changes. The UV-C-HPMC batch showed an increase in luminosity at 9 days; thus, this coating helped reduce the loss of luminosity by up to 18% at the end of storage. The HPMC- LEO-A-P-NP treatment had the same effect on the fresh-cut cucumber, while the UV-C-HPMC-LEO-A-P-NP had an antioxidant effect, which showed that UV-C treatment provided protection as, once again, these were the most stable samples during storage. The color contrast that some fruit showed during storage is another essential feature for consumers. The freshness of cucumbers is largely evaluated by weight loss in the form of drained liquid and the loss of the characteristic light-green color. Associated enzymatic and environmental changes result in the yellowing to which this fruit is susceptible. Figure 2c shows that the Control had the most significant increase in the yellowing index (YI, 71 at day 15) with a rate of increase of 3.98 units/day (R^2^ = 0.97). The HPMC coating reduced the YI to 2.76 units/day (R^2^ = 0.93), thus decreasing product discoloration, as has been reported for other fruits [35,36]. The HPMC-LEO-A-P-NP reduced discoloration during storage to a rate of 1.36 units/day (R^2^ = 0.97), while disinfection with UV-C plus the HPMC- LEO-A-P-NP coating achieved the best color stability, with a change in YI of just 0.72 units/day (R^2^ = 0.93) and a YI value of 37.32. Similar results were documented for fresh-cut cucumber coated with chitosan (2%) and treated in a modified argon-nitrogen atmosphere [26]. The advantage of the present experiment was that the same effect was achieved in a passive atmosphere, as this contributed to the increasing of the shelf life. At 9 days, UV-C had increased the shelf life in the Control samples due to the reduction in microbial growth, and furthermore, UV-C produced cell breakdown with the subsequent increase in oxidation reactions and enzymatic activity, which contributed to a decrease in chlorophyll and changes in the luminosity and yellowing index [31]. However, the treatment with UV-C and HPMC-LEO-A-P-NP helped to prevent oxidation reactions associated with the POD activity, and then stabilized the chlorophylls and color parameters. This behavior is evidenced by the ∆E results and the changes are summarized in Table 2. 

Table 2 shows the ∆E values as a function of storage time and treatment type. It can be clearly observed that the Control samples underwent important changes in color after 3 days of storage. Similar results can be seen for the HPMC coating, with an appreciable color difference at 6 days. In contrast, samples with nano-coating of HPMC-LEO-A-P-NP had their shelf lives increased by at least 9 days; they did not have good control over the microbial growth, which caused appreciable changes in the ∆E. On the other hand, the samples with combined UV-C HPMC-LEO-A-P-NP treatment managed to stay at ∆E < 3 for longer (noticeable difference; at least 12 days) although they still remained acceptable for consumption until 15 days of storage. Thus, combined treatment reduces the reactions associated with microbial growth. It is well known that LEO reduces the oxidation reactions, thereby protecting the product from enzymatic reactions. It acts as a radical donor that prevents the formation of ROS produced by enzymatic and non-enzymatic reactions. The addition of antioxidant nanosystems and ROS homeostasis is considered key in the increasing of shelf life [36,37,38].

### 3.7. Firmness of the Fresh-Cut Cucumber

**Figure 3:**
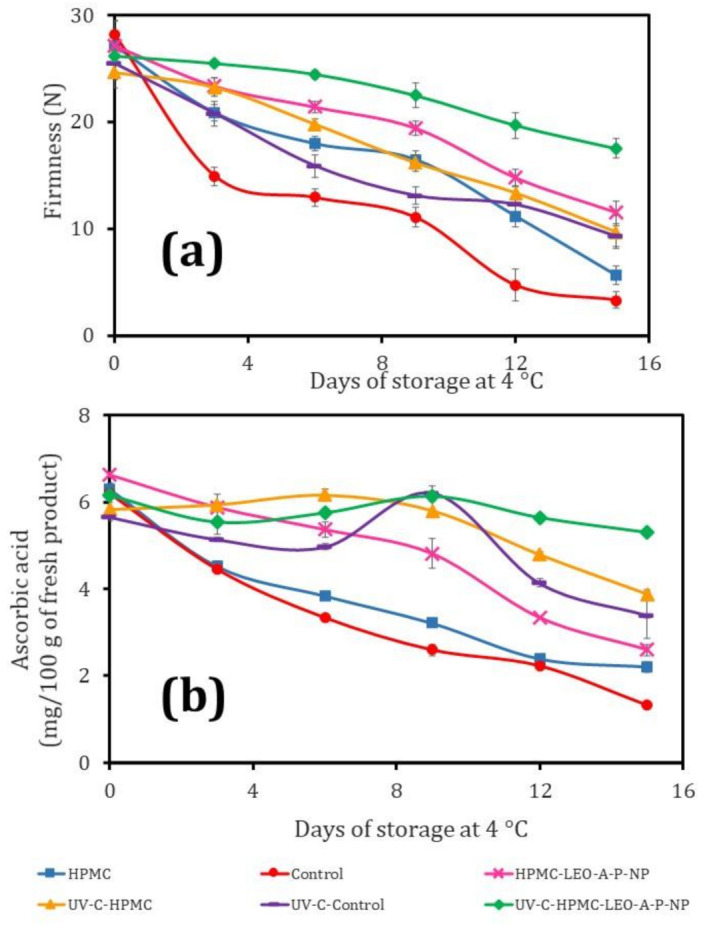
(**a**) Firmness changes in fresh cucumber in relation to storage time at 4 °C; (**b**) ascorbic acid modification in fresh cucumber in relation to storage time at 4 °C.

Firmness is another important sensory aspect for consumers, and is the parameter that is most affected by mechanical operations that can induce the expulsion of intracellular fluid due to tissue rupture [1]. Figure 3a summarizes the changes in firmness for the fresh-cut cucumber. The Control samples had a loss of 47.96% at day 3, with a slight decrease up to day 9, and an index of 86.2% after 15 days. The UV-C-Control batch had better firmness, as the loss was delayed to day 9 of storage (51.5%). The HPMC treatment showed a statistically significant difference (*p* ≤ 0.05) with respect to the Controls. UV-C-HPMC-LEO-A-P-NP and HPMC-LEO-A-P-NP treatment both helped to maintain firmness, with a loss of just 40.41% at day 9. It is important to note that combining HPMC with a beeswax coating had a positive effect on preserving the texture of guava and mango [6,30]. However, the UV-C-HPMC-LEO-A-P-NP had better firmness, with a loss of only 33% after 15 days; thus, the antioxidant and antimicrobial effect of LEO provides an advantage in this regard [39]. UV-C disinfection also positively impacted firmness, as has been reported for such fruits as pineapple and melon [40,41].

### 3.8. Ascorbic Acid Degradation during Storage at 4 °C

Figure 3b shows the AA degradation during storage at 4 °C. The Control lost 58.2% at day 9. In contrast, the HPMC batch decreased by only 40%, and the HPMC-LEO-A-P-NP batch lost just 27.4%. The antioxidants released from the NP (e.g., limonene, phenols) thus provide significant protection [42]. The samples treated with UV-C showed a statistically significant difference (*p* ≤ 0.05) in AA content, as the Control maintained AA up to day 6 and increased it at day 9 due to a metabolic response that affected membrane permeability and generated reactive oxygen species (ROS) and microbial growth. The fresh-cut cucumber disinfected with UV-C and coated with HPMC showed no AA content variation over the first 9 days, but later broke down. The treatment that combined UV-C disinfection with the HPMC-LEO-A-P-NP nano-coating kept the AA content virtually constant during the 15 days of storage, indicating that the LEO released from the NP acted as an oxygen donor to decrease the oxidation of ascorbic acid. These results establish that ascorbic acid decreased less with UV-C treatment [43].

### 3.9. Total Phenol Content

Phenolic compounds are secondary metabolites produced by cells as a defense mechanism. They are released from the cytoplasm during cutting in fresh-cut products, though whole cells respond to external stimuli. Metabolic processes and essential antioxidants and antimicrobials are considered to be bioactive substances in terms of human consumption [7]. Table 3 shows the TPC in the behavior of the fresh-cut cucumber during storage, which was slightly lower (19.6%) in samples without UV-C treatment. This behavior is attributed to the stress the product suffered under a treatment that activates its enzymatic systems. The authors of [31] reported that broccoli subjected to UV-C treatment between 1.5 and 4.5 kJ/m^2^ had an initial increase of 14% in polyphenol content, but this decreased during storage until showing an apparent increase from days 9–12, depending on the coating and UV-C intensity. In the case of the UV-C batch, an increase occurred after 12 days of storage. This behavior matches that reported for broccoli that was also tested at a dose of 4.5 kJ/m^2^, which produced the most significant increase in phenols (54%) after 19 days [31]. The cellular response that induces the enzymatic activity involved in phenylpropanoid metabolism is responsible for the de novo synthesis of phenols [44]. The HPMC-LEO-A-P-NP batch showed a difference between treatment with and without UV-C that was attributable to the antioxidant capacity of LEO. The LEO released during storage had the ability to limit the action of polyphenol oxidases on phenols. The combination of UV-C with HPMC-LEO-A-P-NP helped maintain the product characteristics by limiting the oxidation reaction and controlling enzymatic activity.

### 3.10. DPPH Antioxidant Capacity

Table 3 shows the changes in antioxidant capacity determined based on DPPH activity, expressed as the Trolox equivalent. This treatment showed a statistically significant difference (*p* ≤ 0.05), as the Control with and without UV-C showed the most significant decrease. At the end, these samples showed an increased antioxidant capacity due to the biosynthesis of polyphenols and the oxidation of pigments, which was caused by an increase in the microbial growth [43]. The HPMC-coated cucumber had decreased antioxidant capacity after 9 days of storage, a finding that coincides with the turning point for the changes in other parameters measured in this study. The UV-C-HPMC-LEO-A-P-NP treatment showed a stable antioxidant capacity with a decrease on day 3 followed by a slight increase associated with the release of LEO. Limonene (a major component of LEO) has an antioxidant capacity that helps maintain product quality [45]. The UV-C-HPMC-LEO-A-P-NP treatment showed a slight increase in antioxidant capacity after 3 days. It is important to note that the antioxidant capacity kept the control of microbial growth and deterioration reactions practically constant. The release of LEO from the polysaccharide matrix of the NP, combined with UV-C disinfection, thus helped to increase the shelf life of the fresh-cut cucumber.

### 3.11. Polyphenol Oxidase (PPO) Activity

**Figure 4:**
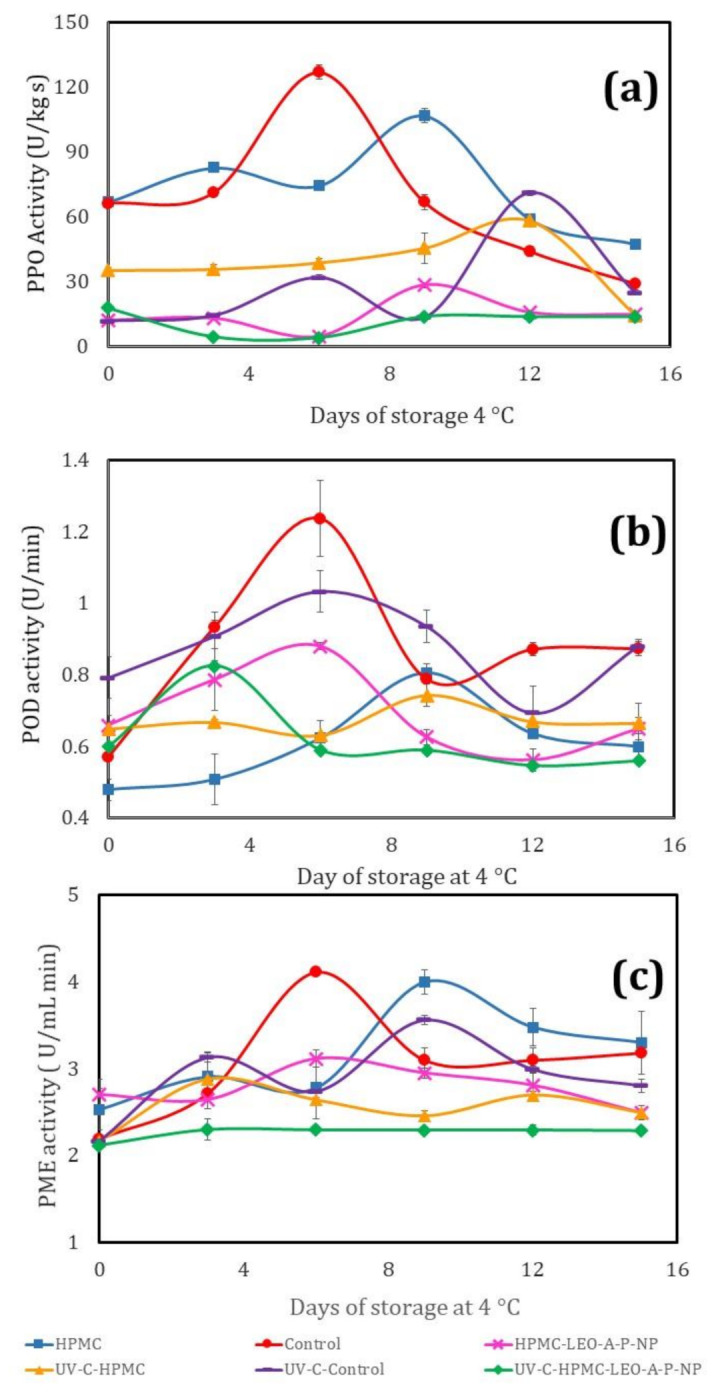
(**a**) PPO activity during storage on fresh-cut cucumber; (**b**) POD activity on fresh-cut cucumber; (**c**) PME activity changes during storage at 4 °C.

Figure 4a shows the changes in PPO activity. In this case, the Control showed maximum activity at day 6 at 127.8 U/kg s; a result that coincides with the decrease in TPC and the color changes that deteriorate product quality. Similar results in PPO activity were reported for fresh-cut cucumber in a study of the effect of ultrasound and carbon-dot coatings [2]. The HPMC coating had only a minimal effect on activity during the first 6 days, followed by a marked increase at day 9. During this interval, these cucumbers suffered an obvious loss of quality that made them unfit for consumption. The UV-C-HPMC batch confirmed that it was possible to delay PPO activity for up to 12 days by a maximum of 58 U/kg s. The combined UV-C-HPMC-LEO-A-P-NP treatment reduced this activity with a notable inhibition of 54%. The reduction in this Control was 43% with respect to the untreated Control. In this regard, the authors of [46] reported that selecting an adequate UV-C dose decreased enzymatic activity in sweet potatoes. A study with fresh-cut apples [47], meanwhile, documented a decrease in PPO activity due to UV-C. In contrast, the fresh-cut cucumber coated with HPMC-LEO-A-P-NP had a considerable decrease in PPO activity with 77.6% inhibition, which helped to conserve both luminosity and color. The UV-C-HPMC-LEO-A-P-NP treatment produced the lowest PPO activity up to a maximum of 15 days, a finding likely related to the antioxidant effect of NP, which shows that antioxidants had a positive effect on PPO activity that decreased enzymatic browning in fresh-cut pears [48]. Our study shows that LEO-A-P-NP presented controlled released during storage, which helped to control deterioration reactions in the fresh-cut cucumber, and that disinfection with UV-C had a synergistic effect that contributed to controlling PPO activity.

### 3.12. Peroxidase Activity (POD)

The principal substrate of the peroxidases consists of phenols which, together with PPO, are responsible for the development of enzymatic browning in fruits and vegetables, including fresh-cut cucumber [48]. Figure 4b shows the POD activity for the different treatments. The Control had more significant activity than any other sample, as it increased by 40% at the end of storage. The UV-C batches had an initial decrease followed by a second decrease during refrigeration, and then an increase after 12 days in all samples. Similar behavior was reported for UV-C-treated papaya stored at 4 °C [49], but it is important to point out that the HPMC-LEO-A-P-NP functioned to decrease POD activity, since those samples underwent only minimal change during storage. It is noteworthy that while the HPMC coating helped protect the fruit, this positive antioxidant effect was reinforced by adding LEO-A-P-NP. The reduction in POD activity was related to the stability of samples during storage, reducing the degradation of chlorophylls and other oxidation reactions associated with the loss of quality [1].

### 3.13. Pectin Methylesterase (PME) Activity

Figure 4c shows the PME activity of fresh-cut cucumber during storage at 4 °C for 15 days. Activity at day 6 was 4.11 U/mL min. This is related to the loss of firmness, which decreased by 54% (Figure 3a. The HPMC coating delayed PME activity up to day 9, confirming that a coating of this kind helps to delay product deterioration, as it coincided with a loss of firmness of just 33% at day 9. In contrast, the LEO-A-P-NP batches had a very slight PME increase at 6 days, which was 24.3% lower than in the Control. This did not vary until day 12. Hence, applying edible coatings (especially ones that include LEO-A-P-NP) can help control PME activity and provide a positive effect on oxidation reactions and the solubilization of pectin in the cell wall, all of which help to maintain product firmness for longer [50]. The UV-C-Control showed an increase in PME activity after 9 days, but this was 14% less than in the batches not treated with UV-C. This behavior was also related to the delay in the loss of firmness. The fresh-cut cucumber coated with HPMC, or HPMC-LEO-A-P-NP, showed the lowest PME activity, though the differences were not statistically significant (*p* ≤ 0.05).

However, treatment with NP that contained LEO presented interactions and protection of cell structures that limited the enzymatic action released by cutting. The antioxidant activity of LEO with UV-C treatment acted on the enzymes to help increase the storage time of the fresh-cut cucumber. A study on the application of UV-C to fresh-cut melon also showed a decrease in PME activity that was more significant with UV-C treatment [51]. It is important to note that the combined treatment had greater effectiveness in reducing PME activity, as the minimum level was observed in the UV-C-HPMC-LEO-A-P-NP batches, where no significant variations (*p* > 0.05) were observed up to day 15.

### 3.14. Microbiological Analyses

**Figure 5:**
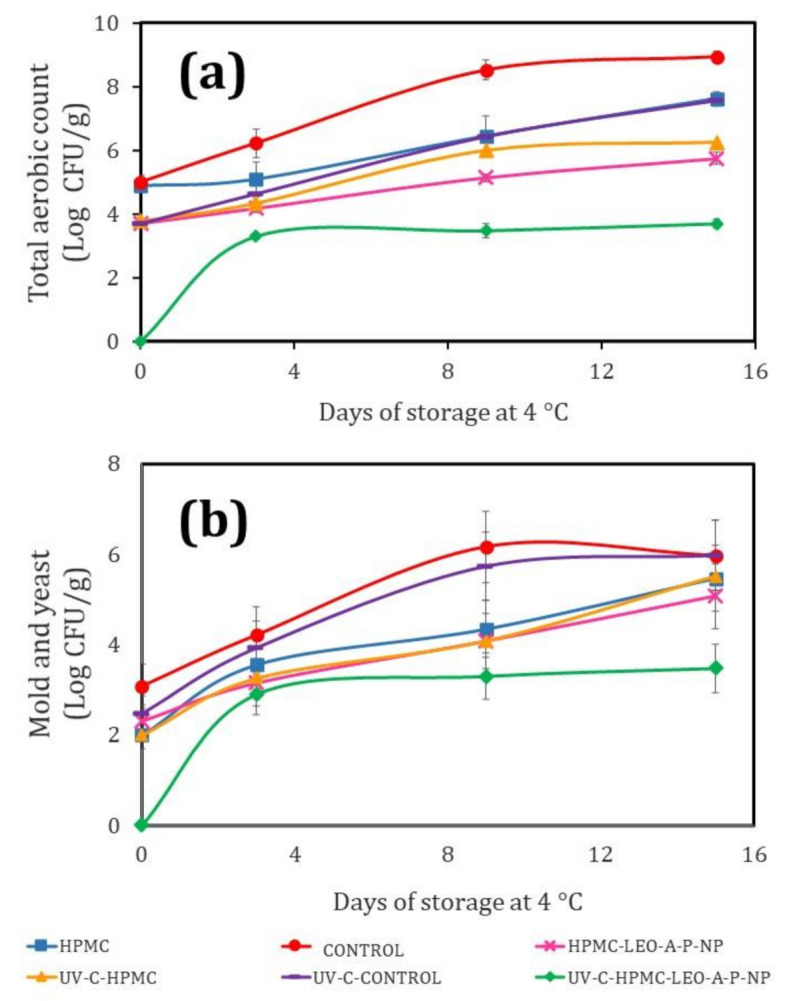
(**a**) Effect of nano-coating on the growth of aerobics and (**b**) mold and fungi on fresh-cut cucumber at 4 °C for 15 days.

Figure 5 shows the effect of the treatments on microbial growth, evaluated by two methods: total count (Figure 5a), and fungi and yeast (Figure 5b).

The total count for the Control showed the highest growth, with a maximum of 8.9 CFU/g. The UV-C-HPMC Control did not show a statistically significant difference (*p* ≤ 0.05). The UV-C-HPMC treatment slightly decreased microbial growth, providing additional evidence that this coating helps to limit gas exchange and reduce the amount of oxygen available for microbial growth. The maximum level reached in this case was 6.24 CFU/g. Significantly, the HPMC-LEO-A-P-NP treatment had a more significant bacteriostatic effect, at a maximum of 5.78 CFU/g. However, the combined treatment (UV-C-HPMC-LEO-A-P-NP) was clearly the best one for controlling microbial growth, as the maximum level determined was just 3.69 CFU/g. A previous [2] study of fresh-cut cucumber coated with chitosan also found that the addition of a film significantly reduced microbial growth. Other studies showed that combining treatments such as modified atmospheres and coatings with biodegradable polymers helps to decrease the growth of *E. coli* [52]. This was true for our combination of UV-C at a dose of 4.5 kJ/m^2^ with HPMC-LEO-A-P-NP, as it reduced bacterial growth and correlated with the other physicochemical characteristics of the product and its conservation. Figure 5b shows fungi and yeast growth during the storage of fresh-cut cucumber. Here, the Control and UV-C-Control fresh-cut cucumbers had the highest growth of fungi and yeasts, at a maximum of 5.93 CFU/g. The treatments with HPMC, UV-C-HPMC, and UVC-HPMC-LEO-A-P-NP, however, did not show any statistically significant differences (*p* ≤ 0.05), indicating that they all reduced fungal growth for up to 9 days, as reflected in the characteristics of product quality.

## 4. Conclusions

In conclusion, it was possible to obtain LEO-A-P-NP with a good encapsulation efficiency and film-forming dispersion that formed a nano-coating on the surface of fresh-cut cucumber. The application of UV-C at 4.5 kJ/m^2^ in combination with a nano-coating of HPMC-LEO-A-P-NP reduced the loss of quality in fresh-cut cucumber. This behavior was associated with the reduction in the initial microbial load caused by UV-C treatment and the protective effect of HPMC-LEO-A-P-NP, which reduced ROS and, hence, the oxidation reactions (both enzymatic and not enzymatic), due to the antioxidant effect of LEO. The application of nano-coatings prepared with natural polymers and essential oil (e.g., lemon) formed a controlled release system with such advantages as eco-friendliness, cost-effectiveness, and ease of application. The hormesis produced by UV-C treatment was compensated with the application of nano-coating. The UV-C treatment limited the microbial growth and helped increase the shelf life of fresh-cut cucumber at 9 days, and the nano-coating of HPMC-LEO-A-P-NP increased the shelf life until 15 days. Moreover, the UV-C modified the initial content of polyphenols and their antioxidant activity. The nano-coating contributed to the stabilization of the enzymatic activity and antioxidant capacity, and was reflected in the quality maintenance of the product.

## Figures and Tables

**Table 1:** SST and pH changes during storage of fresh-cut cucumber at 4 °C.

Days	HPMC	Control	HPMC-NP-LEO-A-P	UV-C-HPMC	UV-C-Control	UV-C/HPMC-NP-A-P
**SST**						
0	3.39 ± 0.20 ^a,a^	3.32 ± 0.31 ^a,a^	2.92 ± 0.32 ^a,b^	3.16 ± 0.21 ^a,a^	3.40 ± 0.10 ^c,a^	2.56 ± 0.21 ^a,c^
3	3.27 ± 0.10 ^a,a^	3.26 ± 0.10 ^a,a^	3.47 ± 0.10 ^b,b^	2.96 ± 0.06 ^b,c^	3.60 ± 0.30 ^a,b^	3.43 ± 0.25 ^b,b^
6	3.19 ± 0.16 ^a,a^	3.16 ± 0.17 ^a,a^	3.57 ± 0.15 ^b,c^	3.24 ± 0.06 ^a,a^	3.40 ± 0.46 ^a,c^	3.43 ± 0.15 ^b,c^
9	3.82 ± 0.23 ^b,a^	3.13 ± 0.10 ^a,b^	3.42 ± 0.16 ^b,c^	2.83 ± 0.25 ^b,d^	3.60 ± 0.55 ^a,a^	3.40 ± 0.26 ^b,a^
12	3.25 ± 0.30 ^a,a^	3.17 ± 0.16 ^a,a^	3.23 ± 0.06 ^c^	3.30 ± 0.17 ^a,a^	3.54 ± 0.51 ^a,b^	3.23 ± 0.15 ^c,a^
15	3.16 ± 0.10 ^a,a^	2.96 ± 0.06 ^b,a^	3.43 ± 0.10 ^b,b^	3.09 ± 0.30 ^a,a^	3.83 ± 0.15 ^b,c^	3.03 ± 0.21 ^d,a^
**pH**						
0	5.41 ± 0.20 ^a,a^	5.26 ± 0.06 ^a,b^	5.19 ± 0.01 ^a,b^	5.10 ± 0.07 ^a,b^	5.35 ± 0.11 ^a,c^	5.19 ± 0.01 ^a,b^
3	5.53 ± 0.13 ^a,a^	5.17 ± 0.05 ^b,b^	5.49 ± 0.02 ^b,a^	5.17 ± 0.02 ^a,b^	5.33 ± 0.10 ^a,c^	5.33 ± 0.05 ^b,c^
6	5.43 ± 0.12 ^a,a^	5.57 ± 0.35 ^c,a^	5.51 ± 0.05 ^b,a^	5.77 ± 0.03 ^b,b^	5.60 ± 0.07 ^b,b^	5.49 ± 0.02 ^b,a^
9	5.59 ± 0.24 ^a,a^	6.24 ± 0.32 ^d,b^	5.48 ± 0.02 ^b,a^	5.61 ± 0.09 ^c,a^	6.29 ± 0.09 ^c,b^	5.54 ± 0.03 ^b,a^
12	6.12 ± 0.19 ^b,a^	6.68 ± 0.08 ^e,b^	5.57 ± 0.02 ^c,c^	5.71 ± 0.12 ^b,c^	6.39 ± 0.17 ^c,d^	5.71 ± 0.10 ^c,c^
15	6.42 ± 0.12 ^c,a^	6.74 ± 0.11 ^e,b^	5.59 ± 0.14 ^c,c^	5.76 ± 0.07 ^b,c^	6.37 ± 0.14 ^c,a^	5.79 ± 0.13 ^c,c^

Different letters to the left represent statistically significant differences (*p* ≤ 0.05) in storage time, and those on the right represent differences in treatment.

**Table 2:** Total color difference (∆E) during storage of fresh-cut cucumber.

Days	Control	HPMC	HPMC-LEO-A-P-NP	UV-C Control	UV-C HPMC	UV-C HPMC- LEO-A-P-NP
0	0.0 ± 0.0 ^a,a^	0.0 ± 0.0 ^a,a^	0.0 ± 0.0 ^a,a^	0.0 ± 0.0 ^a,a^	0.0 ± 0.0 ^a,a^	0.0 ± 0.0 ^a,a^
3	5.1 ± 0.6 ^b,a^	1.8 ± 0.8 ^b,b^	2.1 ± 0.8 ^b,c^	3.8 ± 0.9 ^b,d^	3.9 ± 0.5 ^b,d^	0.8 ± 0.2 ^b,e^
6	9.2 ± 1.2 ^c,a^	6.4 ± 0.8 ^c,a^	2.6 ± 1.0 ^b,b^	7.5 ± 1.2 ^c,c^	5.6 ± 0.9 ^c,b^	1.8 ± 0.5 ^c,d^
9	21.6 ± 1.4 ^d,a^	13.8 ± 1.2 ^d,b^	8.3 ± 1.2 ^d,c^	8.3 ± 1.3 ^c,c^	7.8 ± 0.8 ^d,d^	2.7 ± 0.3 ^d,e^
12	22.8 ± 1.3 ^d,a^	14.8 ± 2.9 ^d,b^	14.3 ± 1.8 ^e,b^	11.8 ± 1.5 ^d,c^	11.8 ± 1.7 ^e,c^	4.1 ± 0.5 ^e,d^
15	26.3 ± 2.3 ^d,a^	21.3 ± 4.3 ^e,a^	17.8 ± 1.6 ^f,b^	27.5 ± 3.6 ^e,a^	20.4 ± 2.1 ^f,b^	7.12 ± 1.2 ^f,c^

Different letters to the left represent statistically significant differences (*p* ≤ 0.05) in storage time, and those to the right represent differences in treatment.

**Table 3:** Total phenols and antioxidant capacity of fresh-cut cucumber.

Days	HPMC	Control	HPMC- LEO-A-P-NP	HPMC-UV-C	Control-UV-C	UV-C-HPMC- LEO-A-P-NP
**Total Phenols (mg EGA/g of fresh product)**						
0	5.83 ± 0.52 ^a,a^	5.79 ± 0.19 ^a,a^	6.36 ± 0.15 ^a,b^	7.44 ± 0.04 ^a,c^	7.12 ± 0.16 ^a,d^	7.80 ± 0.03 ^a,f^
3	5.49 ± 0.24 ^b,a^	5.31 ± 0.18 ^b,b^	6.58 ± 0.20 ^a,c^	5.55 ± 0.06 ^b,a^	4.74 ± 0.44 b,	7.12 ± 0.08 b,
6	5.12 ± 0.34 ^b,a^	2.69 ± 0.08 ^c,b^	6.24 ± 0.10 ^a,c^	5.14 ± 0.10 ^c,a^	3.93 ± 0.03 ^c,d^	6.26 ± 0.09 ^c,c^
9	3.87 ± 0.65 ^c,a^	2.41 ± 0.09 ^c,b^	5.15 ± 0.13 ^b,c^	4.25 ± 0.19 ^d,d^	2.30 ± 0.22 ^d,b^	5.68 ± 0.09 ^d,e^
12	5.78 ± 0.28 ^a,a^	4.96 ± 0.21 ^d,b^	4.88 ± 0.12 ^b,b^	5.35 ± 0.04 ^e,c^	5.14 ± 0.07 ^e,d^	5.62 ± 0.18 ^d,a^
15	3.60 ± 0.99 ^c,a^	5.01 ± 0.23 ^e,b^	7.08 ± 0.03 ^c,c^	6.73 ± 0.83 ^f,d^	6.24 ± 0.59 ^f,d^	4.77 ± 0.42 ^e,b^
**DPPH μmol TE/100 g fresh product**						
0	535 ± 8 ^a,a^	530 ± 12 ^a,a^	567 ± 11 ^a,b^	692 ± 4 ^a,c^	575 ± 17 ^a,b^	657 ± 10 ^a,d^
3	404 ± 16 ^b,a^	399 ± 3 ^b,a^	407 ± 6 ^b,a^	486 ± 2 ^b,b^	415 ± 6 ^b,a^	470 ± 47 ^b,b^
6	327 ± 16 ^c,a^	243 ± 23 ^c,b^	385 ± 25 ^c,c^	339 ± 16 ^c,a^	265 ± 15 ^c,b^	466 ± 17 ^b,d^
9	328 ± 20 ^c,a^	206 ± 8 ^c,b^	405 ± 14 ^b,c^	326 ± 26 ^c,a^	232 ± 41 ^c,b^	482 ± 13 ^b,d^
12	247 ± 33 ^d,a^	335 ± 20 ^d,b^	427 ± 11 ^d,c^	391 ± 18 ^d,d^	209 ± 47 ^c,e^	450 ± 16 ^b,f^
15	460 ± 34 ^e,a^	386 ± 42 ^b,b^	386 ± 51 ^c,b^	326 ± 17 ^c,d^	418 ± 2 ^b,e^	427 ± 3 ^d,f^

Different letters to the left represent statistically significant differences (*p* ≤ 0.05) in storage time, and those to the right represent differences in treatment.

## Data Availability

The data presented in this paper are available upon request from the corresponding author.
